# TOF-PET Image Reconstruction With Multiple Timing Kernels Applied on Cherenkov Radiation in BGO

**DOI:** 10.1109/trpms.2020.3048642

**Published:** 2020-12-31

**Authors:** Nikos Efthimiou, Nicolaus Kratochwil, Stefan Gundacker, Andrea Polesel, Matteo Salomoni, Etiennette Auffray, Marco Pizzichemi

**Affiliations:** Department Radiology, Perelman School of Medicine, University of Pennsylvania, Philadelphia, PA 19104 USA,; PET Research Centre, Faculty of Health Sciences, University of Hull, Hull HU6 7RX, U.K..; University of Vienna, 1010 Vienna, Austria,; European Organization for Nuclear Research, 1211 Geneva, Switzerland.; Department of Physics of Molecular Imaging Systems, Institute for Experimental Molecular Imaging, RWTH Aachen University, 52062 Aachen, Germany,; Physics Department, University of Milano-Bicocca, 20126 Milan, Italy,; European Organization for Nuclear Research, 1211 Geneva, Switzerland.; Physics Department, University of Milano-Bicocca, 20126 Milan, Italy,; European Organization for Nuclear Research, 1211 Geneva, Switzerland.; Physics Department, University of Milano-Bicocca, 20126 Milan, Italy,; European Organization for Nuclear Research, 1211 Geneva, Switzerland.; European Organization for Nuclear Research, 1211 Geneva, Switzerland.; Physics Department, University of Milano-Bicocca, 20126 Milan, Italy,; European Organization for Nuclear Research, 1211 Geneva, Switzerland.

**Keywords:** BGO, Cherenkov, mixture Gaussian model, positron emission tomography (PET), Time-of-Flight (TOF)

## Abstract

Today Time-of-Flight (TOF), in PET scanners, assumes a single, well-defined timing resolution for all events. However, recent BGO–Cherenkov detectors, combining prompt Cherenkov emission and the typical BGO scintillation, can sort events into multiple timing kernels, best described by the Gaussian mixture models. The number of Cherenkov photons detected per event impacts directly the detector time resolution and signal rise time, which can later be used to improve the coincidence timing resolution. This work presents a simulation toolkit which applies multiple timing spreads on the coincident events and an image reconstruction that incorporates this information. A full cylindrical BGO–Cherenkov PET model was compared, in terms of contrast recovery and contrast-to-noise ratio, against an LYSO model with a time resolution of 213 ps. Two reconstruction approaches for the mixture kernels were tested: 1) mixture Gaussian and 2) decomposed simple Gaussian kernels. The decomposed model used the exact mixture component applied during the simulation. Images reconstructed using mixture kernels provided similar mean value and less noise than the decomposed. However, typically, more iterations were needed. Similarly, the LYSO model, with a single TOF kernel, converged faster than the BGO–Cherenkov with multiple kernels. The results indicate that the model complexity slows down convergence. However, due to the higher sensitivity, the contrast-to-noise ratio was 26.4% better for the BGO model.

## I. Introduction

Traditional image reconstruction of data acquired by positron emission tomography (PET) scanners did not include timing information [1]. However, nowadays, it is widely accepted that the incorporation of the Time-of-Flight (TOF) information in the image reconstruction process provides better image properties and improved lesion detectability [2]–[8]. Therefore, manufacturers and researchers alike strive to achieve the best possible timing resolution [9]–[13].

The TOF benefits strongly depend on the timing resolution of the detectors, a crucial component of which is the scintillation crystal. Most scintillation crystals with good timing resolutions are based on the ^176^Lu element [14]–[17]. Compared to Bi_4_Ge_3_O_12_ (BGO), Lu-based crystals present better energy and timing properties; however, to the disadvantage of higher cost, lower stopping power, and presence of intrinsic radioactivity. In addition, BGO’s higher stopping power has a positive impact on the system sensitivity and the parallax effect and hence spatial resolution. Detailed comparisons between Lu-based and BGO crystals have been presented in the past [18], [19].

In recent years, the exploitation of prompt Cherenkov photons observed in some crystals and their potential benefits in improving detection timing abilities has sparked considerable scientific interest, as they provide the prospect of both high sensitivity and compelling timing properties when combined with BGO [20]–[29]. Cherenkov radiation is emitted when charged particles pass through a dielectric medium at speed greater than the phase velocity of light [30]. When measuring with a time-correlated single-photon counting setup and comparing the ratio of prompt light to scintillation light, about 17 ± 3 Cherenkov photons [31] are produced upon 511 keV excitation in BGO.

However, the number of detected Cherenkov photons underlies high fluctuations on an *event-by-event* basis [31], [32]. Therefore, their detection does not provide a single timing resolution but rather a range, which depends on the ratio of the BGO scintillation (slow) to the Cherenkov photons (fast), being detected. The events can then be classified in categories based on the above ratio, with each category having different timing resolution [27].

This article presents the validation of a framework for statistical simulation of full PET scanners with multiple timing spreads. Which, in this instance, we used to approximate the timing performance of the BGO–Cherenkov detectors. Also, we explore the performance of TOF reconstruction with single and multiple mixture timing kernels. Furthermore, we compare the performance of a BGO–Cherenkov scanner model with an LYSO model having a time resolution based on a currently available clinical scanner [33], [34]. Potential benefits in terms of contrast recovery coefficient (CRC), contrast-to-noise ratio (CNR), and noise on image quality using the proposed timing models are presented and discussed. Finally, we briefly discuss some challenges that would have to be addressed to implement a BGO–Cherenkov-based PET scanner.

## II. Materials and Methods

### A. Experimental Determination of the TOF Kernels

The experimental setup for the measurement of the BGO–Cherenkov timing properties has been presented detail previously [32], [35]. In brief, we used 2 × 2 × 20 mm^3^ BGO crystals, wrapped in Teflon and optically coupled with Meltmount to 4 × 4 mm^2^ FBK NUV-HD SiPMs, to measure photon detection time differences from a ^22^Na point source. The signals were readout by high-frequency electronics [35] and digitized by a LeCroy DDA735Zi oscilloscope with 3.5-GHz bandwidth and a sampling rate 20 Gs s^−1^ for each acquisition channel.

**Fig. 1.**
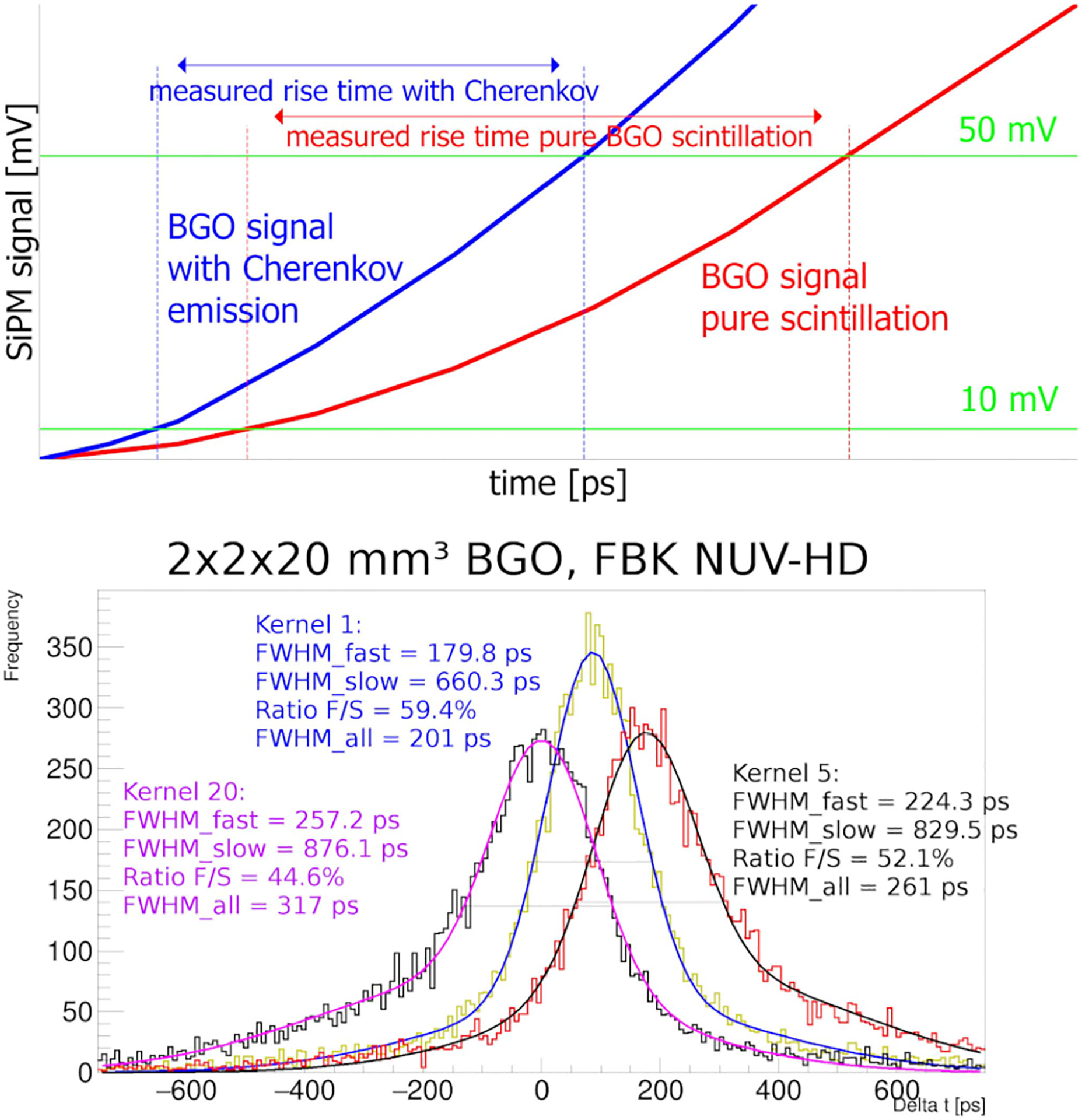
(Top) Principle of the rise time separation: for events where only BGO scintillation is detected the SiPM signal rises slower (red) than the signal where Cherenkov photons are detected (blue) at the beginning of the signal. (Bottom) Time delay histogram with double Gaussian fit function for three categories, where FWHM_fast/slow_ is the FWHM of the fast/slow Gaussian components with abundance ratio *F/S*. FWHM_all_ represents the time resolution of the overall time delay distribution.

We recorded the rise time of the scintillation pulse for every event within energy between 440–665 keV and measured an energy resolution of 19% full width-half maximum (FWHM). The pulse rise times, between 10 mV and 50 mV, together with the coincidence time delay were stored and analyzed offline. Events with a large number of detected Cherenkov photons had faster signal rises, and we classified them based on it. This classification is illustrated on Fig. 1(top).

We partitioned the events in 5 categories per detector based on the measured signal rise time. Each category contained 20% of the photopeak events (4% of the events in coincidence). The distribution of the time delays was modeled with a double Gaussian mixture model [31].

The time resolutions ranged from 201 ps FWHM (544 ps Full Width-Tenth Maximum (FWTM)) to 333 ps FWHM (1109 ps FWTM), depending on the category. Merging all categories, the system had a timing resolution of 288 ps FWHM (954 ps FWTM) before time-walk correction, and 259 ps FWHM (891 ps FWTM) after [27]. The coincidence time delay of three of the categories is shown in Fig. 1(bottom). More details on the experimental measurement, analysis and results can be found in [27].

### B. Simulation Toolkit

We performed the scanner simulations on a modified GATE Monte Carlo (MC) simulation toolkit (v8.1) [36]. In particular, we modified the GateTemporalResolution and GateCoincidenceSorter classes. Initially, they apply the timing spread on each single *γ*-photon, individually. However, to accurately reproduce the measured timing kernels, we applied the time spread in the GateCoincidenceSorter, a class that pairs the singles to coincidences.

For that, we generate a random number using a uniform probability density function (PDF) and the G4RandGeneral.shoot() function. We choose which of the 25 timing categories (rows of Table I) to apply on the event pair, based on this number. Then, we generate a second random number, using the same method, which is compared with the abundance ratio (*α*_*p*_) (Table I column 3) to choose either the fast or slow component. The selected time spread is applied on the two single timestamps selecting a random number from a normal PDF with the specified distribution. Finally, before storage, the pair is checked for coincidence. The information on which timing spread and component were applied is stored in the output files.

#### 1) Scanner Simulation Models:

All scanner models in this study have the same geometry. They have 666 crystals per ring, each with the size of 4 × 4 × 20 mm^3^, arranged on 48 rings with an axial length of ≈ 20 cm. The geometry was designed to be as close to a cylinder as possible, avoiding any blocks and gaps. The scanner models vary in crystal material (BGO and LYSO), timing and energy properties, and coincidence window.

The BGO scanner model was simulated with two timing spreads: 1) BGO-Ch used a single double-Gaussian mixture model (179.8 ps and 660.3 ps) (first row of Table I) and 2) BGO-mCh with 25 mixture timing spreads, as summarized in Table I. Both BGO scanner models had 19% energy resolution, energy window 425–650 keV, and coincidence window to 9.0 ns.

The second scanner model had LYSO crystals (density: 7.105 g.cm^−3^), 11% energy, and 213 ps timing resolution. The values approximate those reported for the Siemens Vision PET scanner [33], [34]. The coincidence window was 4.0 ns, and the energy window was set to 450 – 650 keV. The minimum radial distance for two singles to be considered for coincidence was set to 83 r-sectors (as defined in GATE).

We should note that the BGO crystal profiles in the simulation are not the same as those in the experiment. It is expected that crystals with a profile (4 × 4 × 20 mm^3^) will have slightly poorer timing properties. The deterioration with larger crystal cross-section (3 × 3 × 15- instead of 2 × 2 × 15 mm^3^) was measured to be about 8% [28].

#### 2) Simulated Acquisitions:

We simulated five noise realizations of the NEMA image quality (IQ) phantom [37] with background activity 11.38 kBq/cc (42 MBq total activity), and hot spheres ratio 4:1. The source emitted back-to-back photons. The two larger spheres were made of water and left without activity. The simulated acquisition time was 150 s for all models. We considered one bed position without the scatter cylinder. We did not include a bed in the simulations. The phantom spheres were surrounded by a cold region representing the container’s wall; the inner diameter was 10, 13, 17, 22, 28, and 37 mm.

In order to compare the performance of the multiple mixture kernels with the single typical Gaussian kernels, on a similar number of events, a complementary dataset (BGO-mCh (low)), was generated as a subset of the BGO-mCh events.

### C. Image Reconstruction Toolkit

We used software for tomographic image reconstruction (STIR) (v.4.0) [38], [39] for the reconstruction of the simulated data, using TOF listmode maximum likelihood-expectation maximization (LM-MLEM) as it is the most robust option and is guaranteed to converge to a solution [40]. The validation of the TOF reconstruction with Gaussian [40]–[42] and non-Gaussian [43] kernels has been presented in detail previously. In this study, the application of the complex TOF kernel is done similar to that of the simple Gaussian. In brief, first, we calculate the non-TOF line-of-response (LOR) (*p*_*ij*_) and then the timing kernel is applied as

(1) $$p_{it;jp}=p_{ij}K_{it;jp}$$

(2) $$K_{it;jp}=\left(1-\alpha_{p}\right){\left({\text{cdf}}_{\text{S}}\left(k_{t+1}-v_{cj}^{\prime }\right)-{\text{cdf}}_{\text{S}}\left(k_{t}-v_{cj}^{\prime }\right)\right)}_{p}+\alpha_{p}{\left({\text{cdf}}_{\text{F}}\left(k_{t+1}-v_{cj}^{\prime }\right)-{\text{cdf}}_{\text{F}}\left(k_{t}-v_{cj}^{\prime }\right)\right)}_{p}$$

where *K*_*it*;*jp*_ is the time response for the *t*th TOF position of the *i*th bin and *j*th image element, cdf_*F*_ and cdf_*S*_ are the cumulative distribution function (CDF) corresponding to the fast and slow components of the Gaussian mixtures, [*k*_*t*_, *k*_*t*+1_) is the timing interval for the *t*th TOF bin and $v_{cj}^{\prime }$ is the projection of the voxel’s center on the TOF bin, *p* denotes the timing kernel for the particular event (1 to 25), and *α*_*p*_ is the corresponding abundance ratio between the two components (ratio F/S in Table I).

#### 1) Image Reconstruction:

The reconstruction was performed for 150 iterations, although most discussion and analysis was focused on the 40th iteration, which has been used for Siemens Biograph Vision [34], evaluation and is an acceptable choice in the clinic.

An image grid of 320 × 320 × 95 voxels with size 1 × 1 × 2.08 mm^3^ was used. The size of the TOF bin was set to 1 ps. No TOF mashing, view mashing, or axial compression was used at the data. Furthermore, post-filtering was not applied on the reconstructed images.

The attenuation correction factors were calculated with an analytical projection of a digital representation of the emission phantom, having the appropriate linear attenuation values for 511 keV *γ*-photons, as found in NIST [44]. Post reconstruction, the images were calibrated to units of activity concentration (kBq/cc), by scaling the mean background value of the 150th iteration.

The performance of background correction methods can vary widely [45], the detectors under consideration are novel and in many aspects, unique. Therefore, in this study, we focused on demonstrating the potential benefits of the multiple timing kernels and higher sensitivity, using only true coincidences.

#### 2) Reconstruction Models:

The simulation with the multiple double Gaussian mixture timing spreads was reconstructed using the same 25 mixture kernels (BGO-mCh), as summarized in Table I. The simulation with the single double-Gaussian mixture spread (BGO-Ch) was reconstructed using two different approaches. In the first case we used a mixture TOF kernel (BGO-Ch-mix). In the second, we used the same single Gaussian components (fast or slow) that was applied on each coincidence pair during the simulation (columns 2 or 3 Table I). Essentially, we decompose the mixture kernel in two simple Gaussian kernels (BGO-Ch-dcmp).

It has been suggested in the literature that the use of mixture kernels in maximum likelihood-expectation maximization (MLEM) does not lead to the optimal result. Whenever possible simple Gaussian kernels should be used [46]. The LYSO scanner was simulated with a typical Gaussian spread, therefore, a typical Gaussian TOF kernel (213 ps FWHM) was used in the reconstruction. Finally, for comparison, we included a non-TOF reconstruction.

**Fig. 2.**
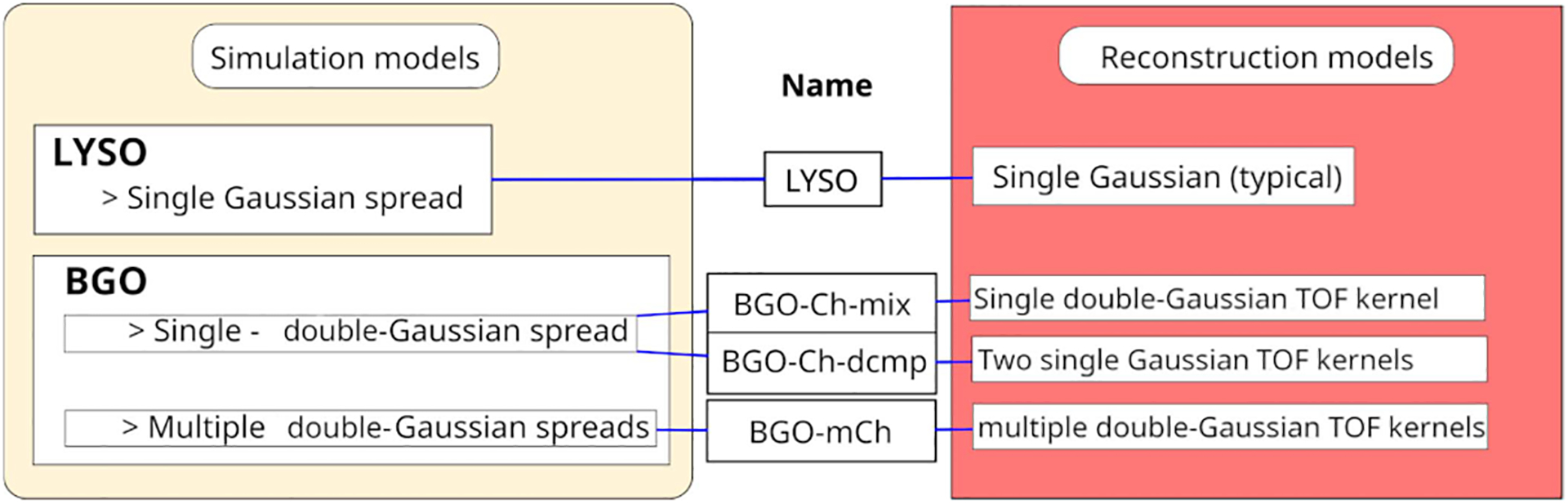
Overview of the simulation and reconstruction models. The single double-Gaussian mixture spread used the values from the first row of Table I.

Simulation cases and reconstruction models are summarized in Fig. 2.

### D. Regions-of-Interest and Figures-of-Merit

In the calculation of CRC and background variability (BV), we followed the 2012 NEMA guidelines [37]. In brief, for each sphere (*r*), we drew circular Region-of-Interest (ROI)’s, on the central phantom slice, having the same diameter as the sphere. We sampled the background with 60 equally sized ROIs for each sphere. The same ROIs were used for the presentation of the standard error (SE) and ROI mean value.

We evaluated the CNR following the methodology suggested previously [47] using the Regions-of-Interest (ROIs) from the contrast calculation and the following formula:

(3) $${\text{CNR}}_{r}=\frac{\mu_{H,r}-\mu_{B,r}}{\sqrt{\sigma_{H,r}^{2}+\sigma_{B,r}^{2}}}$$

where *μ*_*H*,*r*_ is mean values of a circular ROI, over sphere *r*, $\mu_{B_{r}}$, and *sigma*_*B*,*r*_ are the mean value and average standard deviation (SD) at the associated background regions.

The bias from each reconstruction method is evaluated according to a methodology adapted from literature [48]. In this section, in order to avoid the partial volume effects, each ROI has half the diameter of the corresponding sphere. Each ROI’s statistics are calculated across the multiple noise realizations and are compared to the expected activity concentration.

## III. Results

### A. Accuracy of Simulation and Statistics

**Fig. 3.**
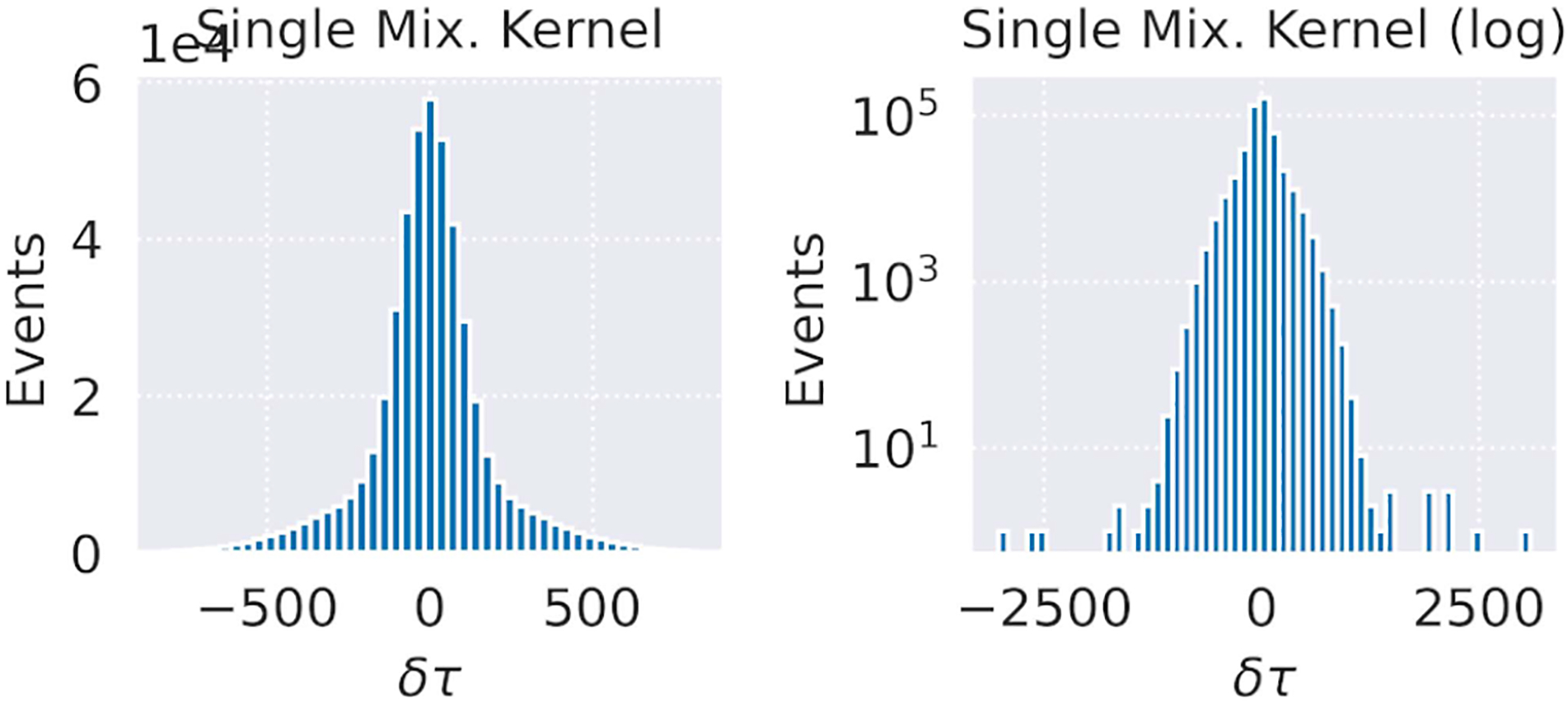
Demonstration of the simulated time differences, using the modified GATE toolkit. The source was a point at the center of the FOV. (Left) Time distribution from a single double-Gaussian mixture spread (13th row of Table I) and (right) same on a log-scale.

In Fig. 3, we demonstrate the simulated time difference histogram of a point source placed at the center of the field-of-view (FOV). In this simulation, we used only the timing values of row 13 of Table I.

**Fig. 4.**
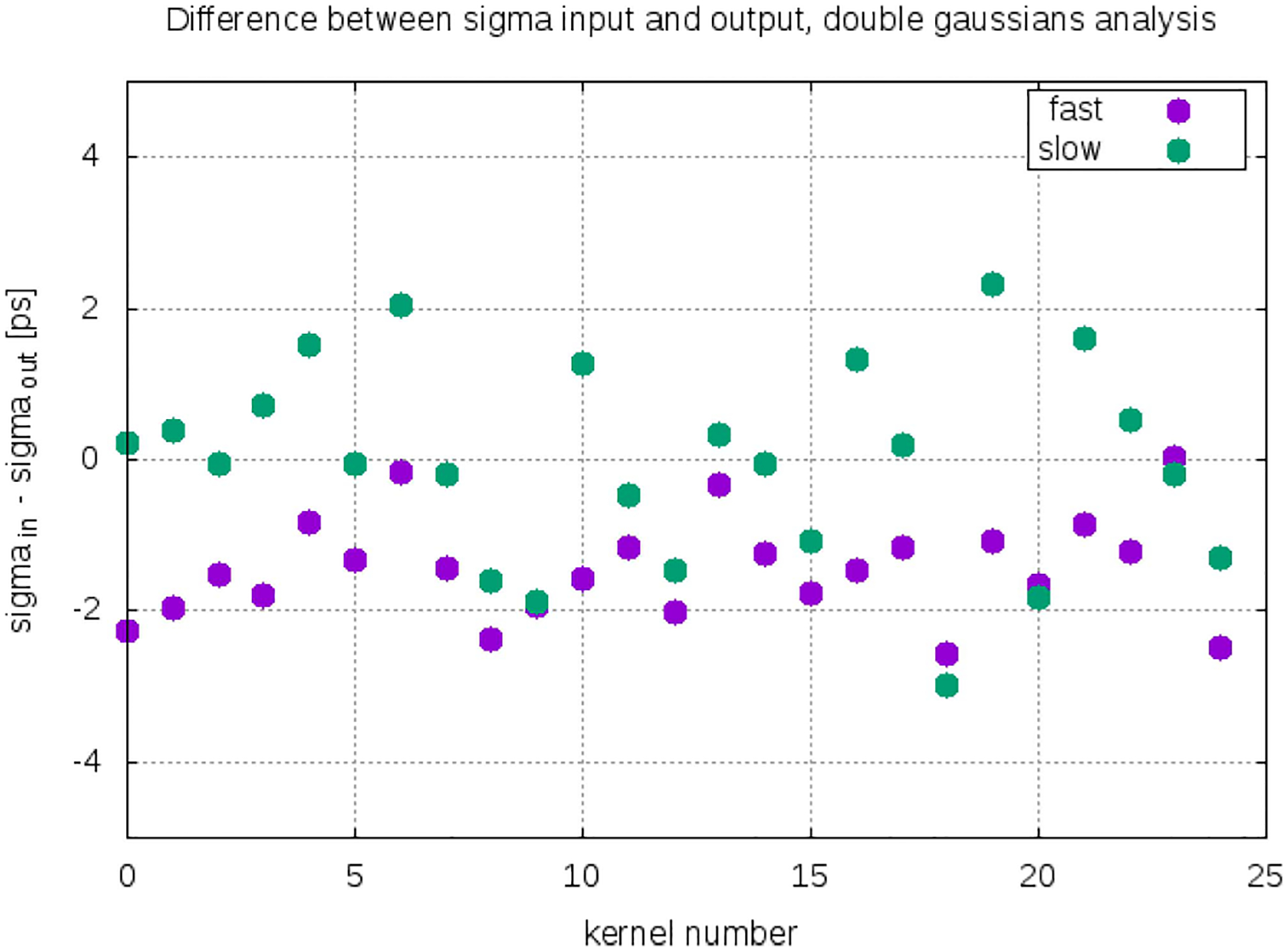
Difference between simulated and experimental sigma of each of the 25 timing categories given at Table I.

Fig. 4 shows the difference between the sigma of the experimentally measured kernels (input) and the simulated (output) for every row of Table I. The data show that the simulated timing kernels are in good agreement with the experimental. There is an approximately ±2 ps error which can be attributed to the statistics of the simulation.

With the NEMA phantom, the BGO model recorded on average 69.2 × 10^6^ coincidences. The ratios Trues/Prompts, Randoms/Prompts, and Scattered/Trues were 56.92%, 19.67%, and 41.1%, respectively. The LYSO model recorded 38.0 × 10^6^ coincidences and the corresponding ratios were 67.9%, 10.4%, and 31.76%. The above ratios demonstrate the negative impact of the BGO’s energy resolution and larger coincidence window.

However, for the image reconstruction, we used only true events and the BGO model recorded about 34% more than the LYSO; 39.37 × 10^6^, 39.37 × 10^6^, and 25.88 × 10^6^ for the multiple kernel, the single kernel BGO (and the non TOF), and the LYSO models, respectively. The complementary BGO-mCh-low dataset had on average 26.27 × 10^6^ true events.

We did not observe a significant difference in the total number of detected or true events between repetitions. We should note that multiple coincidences were left out of the simulation, as our multiple timing resolutions approach could potentially lead to the application of different timing spreads on pairs sharing one common single event, e.g., the same single being classified as fast for one pair and slow for another pair.

### B. Mean Value and Standard Error

**Fig. 5.**
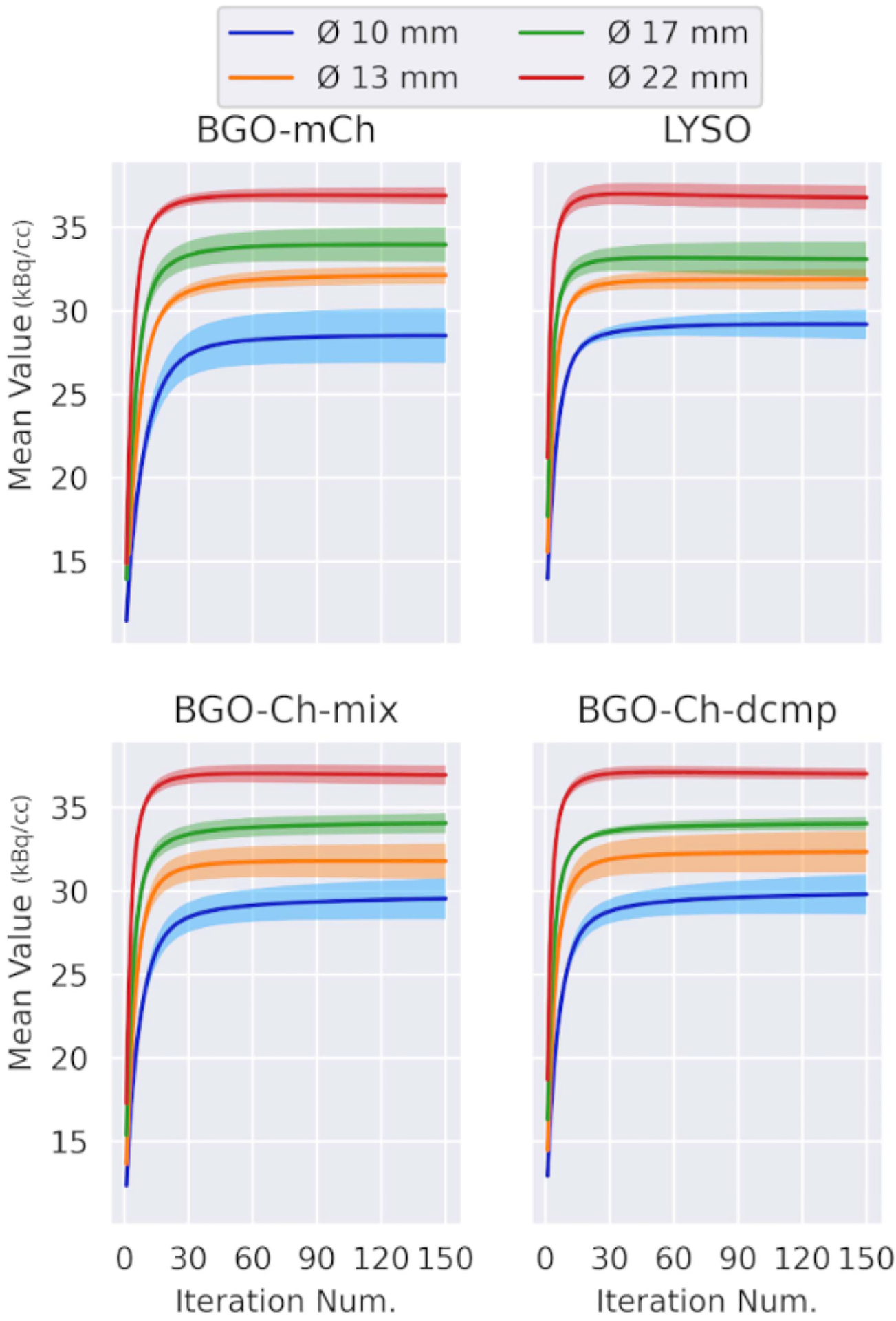
Mean value of the four hot spheres for every TOF model. The error bars are standard error over the five noise realizations. The BGO-Ch-mix and BGO-Ch-dcmp were reconstructed from the same dataset. BGO-mCh-low is a subset of BGO-mCh, with count number similar to that of LYSO.

In Fig. 5, the mean value and SE of each hot sphere and all TOF models are presented. We see that the spheres converge with different rates for each reconstruction model. The mean of the 22-mm sphere reached a roughly constant value on the 80th, 61th and 54th and 37th for the BGO-mCh, the BGO-Ch-mix, the BGO-Ch-dcmp, and LYSO, respectively. As the results suggest that the single Gaussian model converges with a faster pace than the more complex models.

The BGO-Ch-mix and BGO-Ch-dcmp, which share the same simulated dataset, showed that the exact Gaussian component (BGO-Ch-dcmp) achieves convergence earlier than the use of the mixture model (BGO-Ch-mix). In addition, decomposition provided much smaller SE at the two larger spheres and higher mean value for the smaller sphere. However, BGO-Ch-dcmp performed worse with the 13-mm sphere in terms of SE.

Overall, the complexity of the timing model seems to have a negative impact to the convergence rate. However, the differences are not strong.

### C. ROI Statistics

**Fig. 6.**
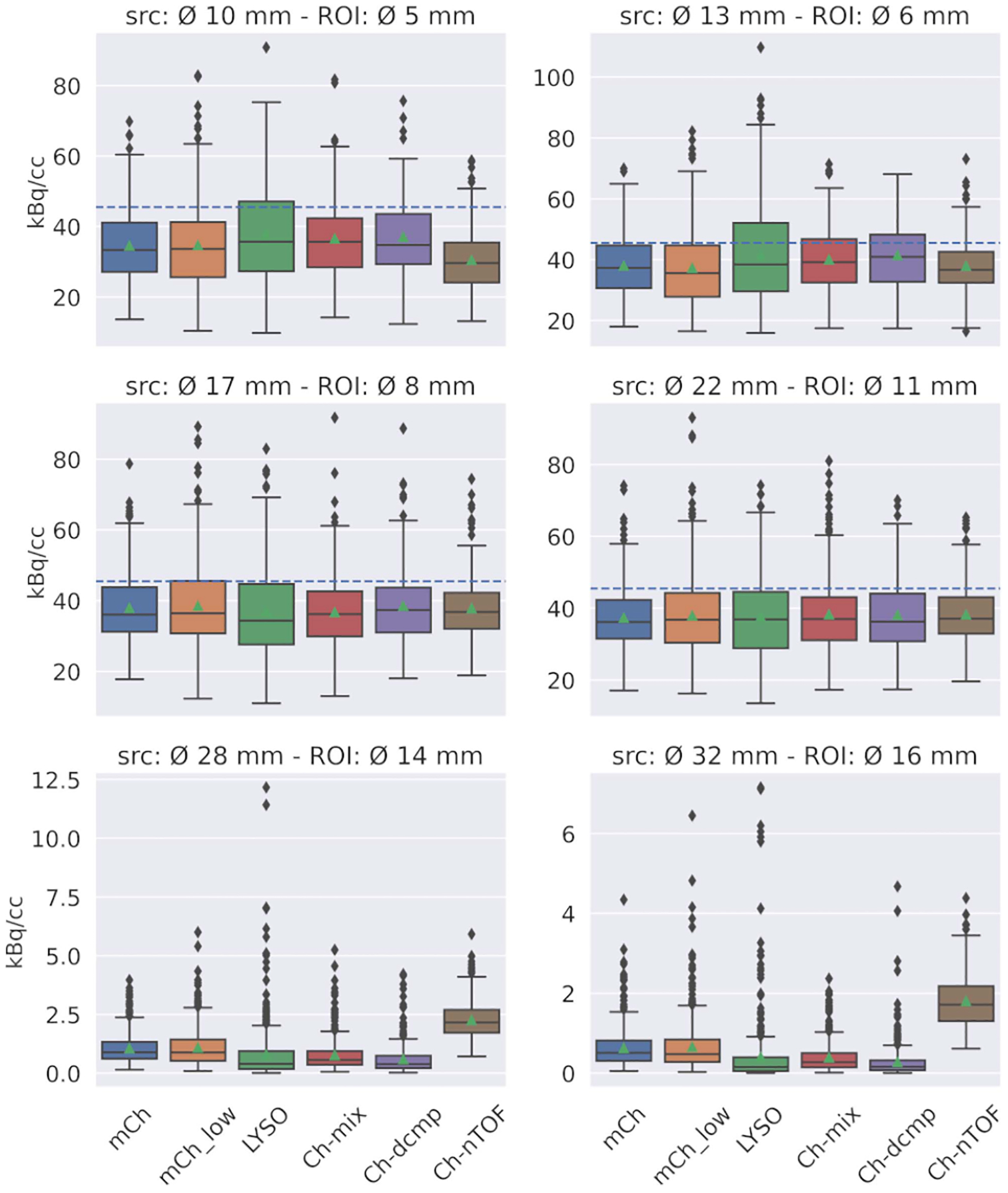
Distribution of activity concentration values from the central part of all spheres in the NEMA-IQ phantom on the 40th iteration and across five noise realizations. The expected activity concentration is indicated with a horizontal line.

In Fig. 6, the distribution of voxel values for the central part of each ROI, across all five noise realizations, on the 40th iteration, is summarized. We see that reconstruction with single Gaussian (LYSO) yields a smaller value density than single or multiple, double-Gaussian mixture kernels, even for a similar amount of events. The previous observation is more marked for the smaller sources. However, the median values are similar indicating, a right side skewed distribution, which, the complex TOF models, presented in a much lesser degree. The skewed distribution might be a result of noise amplification and non-negativity constrains and lead to the LYSO’s mean value be closer to the expected.

Surprisingly, in the host spheres, we cannot detect a significant difference, in terms of mean value, between the use of mixture and decomposed TOF kernels. Although the latter has access to more information, and in the previous paragraph, we showed that it converges sooner. However, we see a lower value density, which indicates higher noise and maybe is further in the reconstruction process. As expected, the non-TOF has the lowest mean value in the smaller spheres as it needs to be iterated more.

In the cold spheres, we see the single Gaussian model to have the lowest median value and better mean value (closer to 0) than the models with multiple mixture kernels. Moreover, the decomposed model showed denser value distribution and better mean and median values, than the mixture model. As was the case in the host spheres, the non-TOF lacks in convergence compared to the TOF models.

**Fig. 7.**
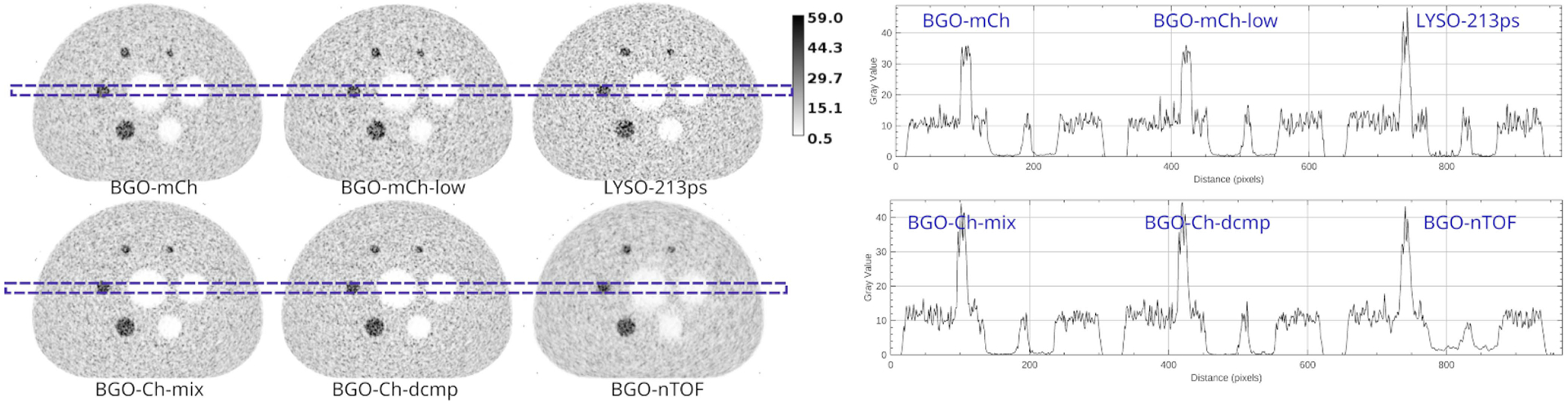
Reconstructed images at the 40th iteration for the different detector models for one of the noise realizations.

In Fig. 7, we demonstrate the reconstructed central slice and associated line profiles (average of five rows) of all cases on the 40th iteration, from one of the noise realizations. One can see the increased noise in LYSO (background and source) and possibly notice a slightly noisier BGO-Ch-dcmp in comparison to the BGO-Ch-mix. In addition, LYSO has sharper boundaries than BGO around cold regions.

### D. Contrast Recovery

**Fig. 8.**
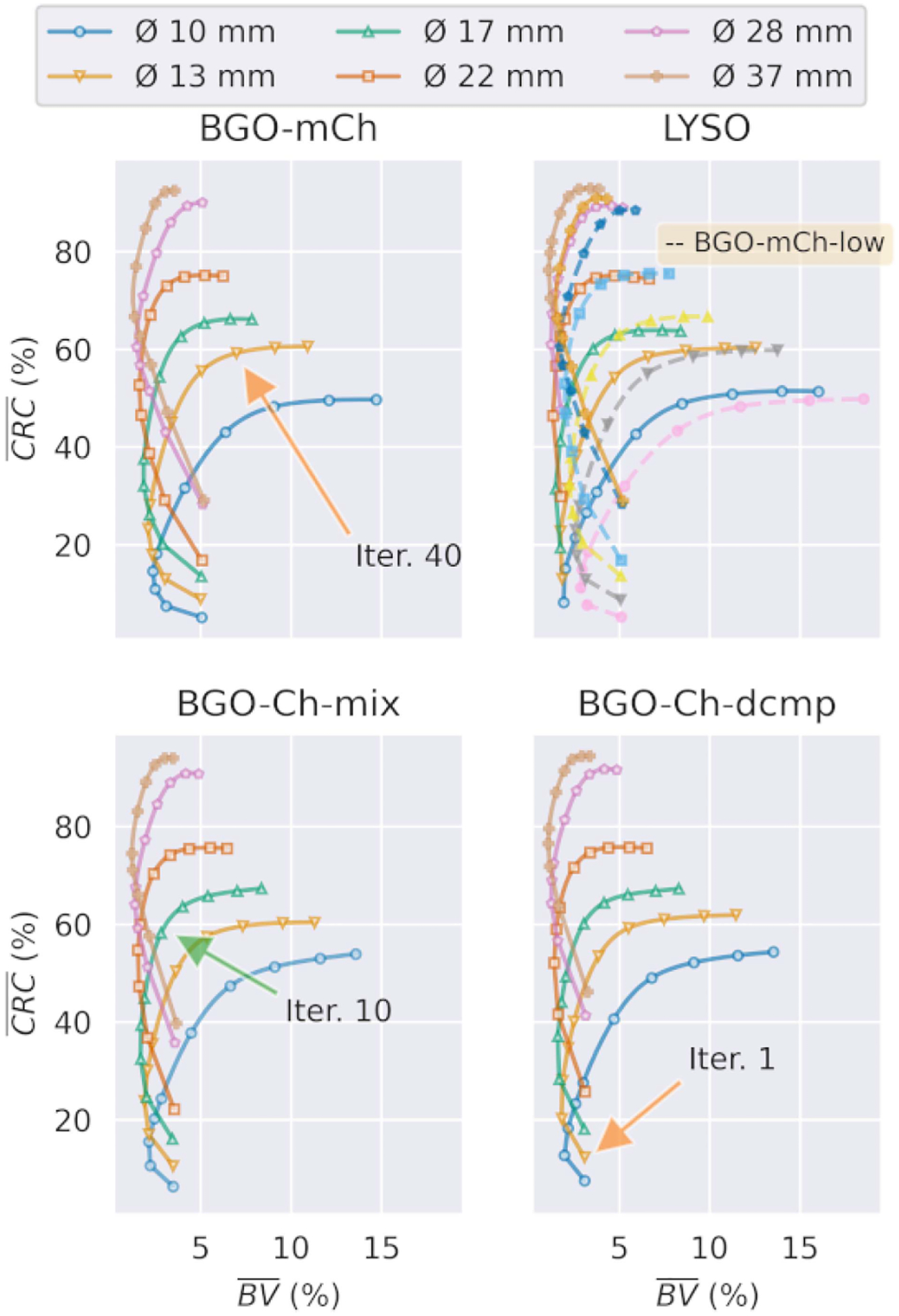
Contrast recovery coefficient for every hot and cold ROI in the NEMA phantom for the multiple double-Gaussian mixture (BGO-mCh), single double-Gaussian mixture (BGO-Ch-mix), single double-Gaussian decomposed (BGO-Ch-dcmp), and single Gaussian-213 ps (Gaussian) models. Along side the LYSO model, with dashed lines the low count BGO-mCh-low, is given. The markers annotate iteration numbers: 1, 2, 3, 4, 5, 10, 20, 40, 80, and 150.

Fig. 8 shows the average contrast recovery (*CRC*) against the average BV (*BV*), averaged over the five noise realizations. The markers on the curves indicate the iterations with number 1, 2, 3, 4, 5, 10, 20, 40, 80, and 150.

Noteworthy is the reduction in *BV* that takes place in the first five iterations for the complex timing models. The above behavior is more marked in the BGO-mCh case, and to a lesser degree in the BGO-Ch-mix and BGO-Ch-dcmp models. Also, the mixture-kernels’ recovery during the first iterations is relatively lower than that of the single kernel. Result of this *slow start* is that the mixture-kernel’s convergence step becomes larger, during iterations from 10 to 40 than that of the regular TOF reconstruction. In most cases, the mixture-kernels’ *CRC* reaches a similar level with the single kernel by the 40th iteration.

Comparison between LYSO and BGO-mCh-low, which had a similar number of events, at the 40th iteration shows that the *BV* is comparable. However, at later iterations, the simple Gaussian model manages noise more efficiently.

Comparison of the Ch-mix and Ch-dcmp models (which use the same datasets) shows that using the decomposed kernel benefits the contrast recovery during early iterations, as in has higher *CRC*. However, after the 30th iteration, the mixture model has similar recovery with slightly better *BV*.

### E. Contrast-to-Noise Ratio

**Fig. 9.**
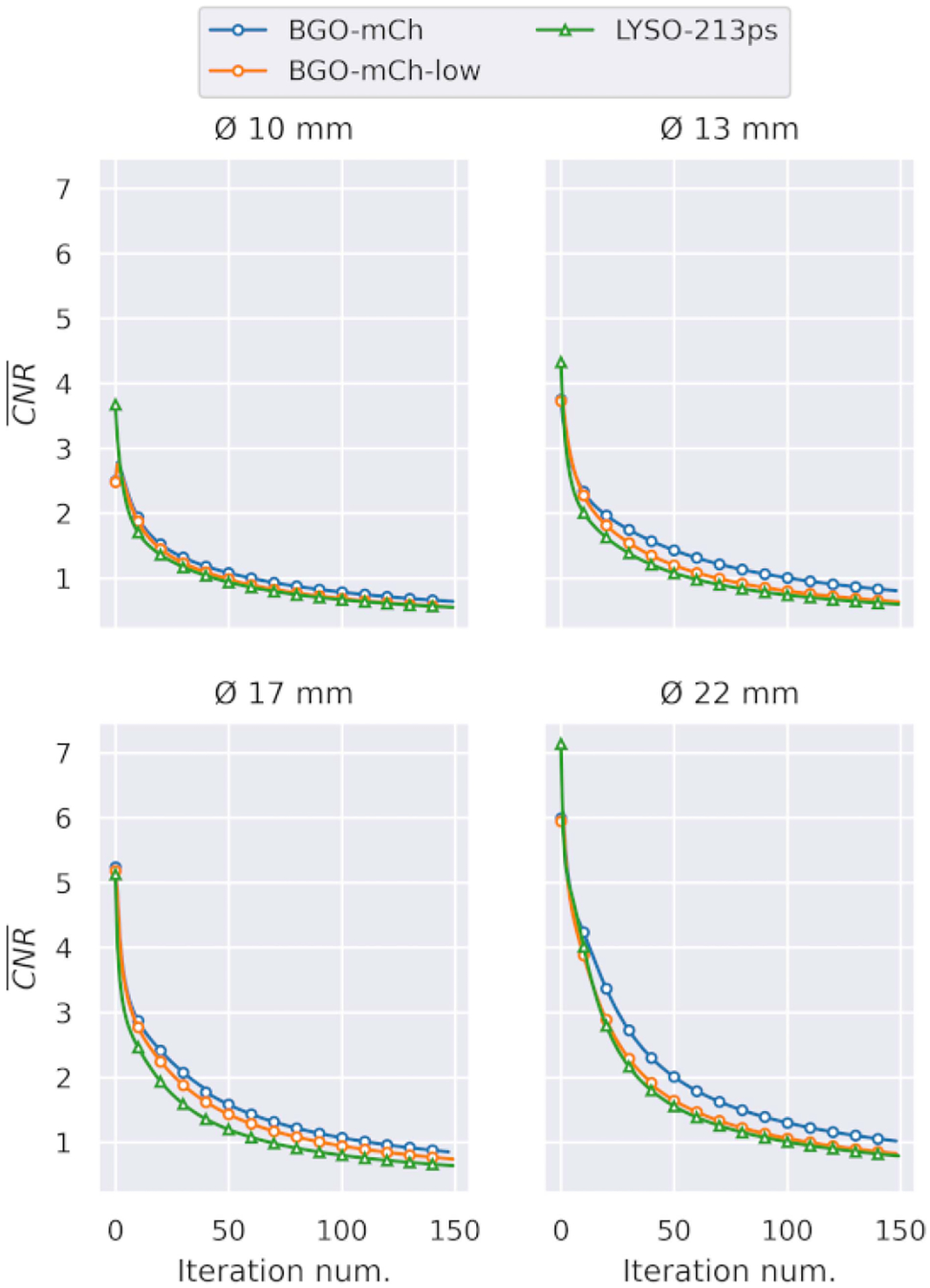
CNR for all hot spheres of the NEMA phantom using the BGO with multiple double-Gaussian mixture kernels and two count levels and an LYSO reconstructed with single Gaussian kernel.

Investigating the *CNR iteration-after-iteration*, we can see that the higher sensitivity of the BGO scanner has a strong positive impact (Fig. 9). We report up to 22.79% higher *CNR* for the 22-mm sphere and about 26.40% for the 13 mm. In 10 mm, the difference CNR is similar between the two models, primarily due to the low contrast. The BGO-mCh-low performance was in-between the BGO-mCh and LYSO models, depending on the size of the source. The difference in the performance of the BGO-mCh-low can be explained by the high SE that some sources demonstrated (Fig. 5).

## IV. Discussion

This article presented the validation and initial evaluation of a Monte Carlo (MC) framework for the statistical simulation of data modeled for detectors that produce multiple, complex, time resolutions. In this instance, we used the framework to investigate BGO–Cherenkov detectors, modeled after experimental measurements. Previously, we demonstrated that the timing of BGO–Cherenkov detectors can be modeled with multiple double-Gaussian mixture kernels. The different kernels account for a different number of Cherenkov photons detected the pulse rise time, alongside the normal BGO scintillation light.

We based the MC simulation framework on the GATE toolkit that we appropriately modified. Unlike previously published methods for the simulation of Cherenkov photons [49], [50], we used a statistical approach which apply multiple timing spreads on photon pairs considered for coincidence.

Simulated timing histograms from a point source in the center of the FOV of a generic cylindrical PET showed excellent agreement with the experiments (±2 ps). The repeated simulations with the NEMA-IQ phantom showed that the BGO crystal measured approx. 34% more coincidences than LYSO, due to its high density. However, had poorer scattered ratio, due to the lower energy resolution. In the activity level used in this investigation, the difference in randoms was not large.

Also, we investigated the effect of reconstruction with multiple TOF kernels in the PET image quality. Comparison between the BGO-mCh model (BGO detectors, multiple mixture kernels) with the LYSO (single Gaussian kernel) showed that the former is able to achieve similar CRC (after the 40th iteration) with lower BV. The advantages of the higher stopping power are demonstrated in terms of CNR with an improvement of 26.4%. The smaller SD (not shown here) in the ROIs using BGO can potentially benefit other metrics, as the max standard uptake value.

For a comparable number of events (case of BGO-mCh-low), the simple Gaussian kernel performed better in contrast, at iterations below 40th and, in terms of noise after the 70th (subject to the size of the sphere). Having that said, the difference was not big under visual inspection. Therefore, the use of BGO detectors with TOF could lead to lower administered doses or shorter acquisition time.

Comparison between BGO-Ch-mix and BGO-Ch-dcmp showed that the exact timing model offers a slightly better performance in terms of mean value and standard error. However, second-order metrics (i.e., CRC and BV) did not show strong differences. Meaning that the lack of this information is not detrimental to the quality of the reconstructed images.

Using multiple mixture Gaussian TOF kernels, we observed an initial reduction in the BV. This is an unusual behavior that has been demonstrated in the past when using shift-invariant [51] and shift-variant [52], image-space point spread function (PSF) correction. A possible explanation for this behavior is the different distribution of timing resolutions within each background ROI. Therefore, each background ROI initially has a different convergence rate and noise level, leading to a relative bias, which the algorithm tries to minimize. However, after a few iterations, approximately 5, the dataset’s noise dominates the standard deviation, and the BV starts increasing.

As a general comment on the complexity of the timing model, the results suggest a negative effect on the performance of MLEM. Maybe categorizing the events in fewer categories, or use of different algorithms could improve upon that. However, as shown by CNR, it is a *price worth paying* for the benefit of the higher sensitivity.

This study has several limitations, which have been discussed throughout the manuscript, but we will try to summarize here. In brief, full BGO scanners with fast acquisition electronics and one-to-one coupling have not been manufactured in the past. Therefore, we did not have a precedent to base our simulation model. To confirm the used energy resolution, we performed a dedicated light yield measurement with ^137^Cs and a PMT from Hamamatsu (R 2059) and measured an energy resolution of 17.3% for 2 × 2 × 20 mm^3^ crystal geometry. Scaling this value down to 511 keV leads to an energy resolution of 19.6%, which was not ideal.

As we cannot accurately predict a full BGO-Cherenkov PET scanner’s potential performance characteristics, we chose to omit the background events and focus on reconstruction of true events only, which limits the presented findings to a certain degree. Other effects as dead-time, pile-up, etc., will probably have to be addressed in future works.

A final point that has not been discussed in detail is illustrated in Fig 1; the mean value of the measured timing distributions is not centered at zero. It happens as the two pulses may not pass the lower threshold simultaneously due to the different rise times. In the NEMA investigation, we omitted this shift by setting the simulated kernels to be centered. Like the TOF bin time shift, a correction algorithm needs to be developed and applied in a simulation and the physical scanner.

## V. Conclusion

In this article, we presented a validation and a performance evaluation of a framework that can be used to simulate and reconstruct data from TOF PET scanner geometries, modeling BGO–Cherenkov detectors. The reconstruction considers the multiple timing resolutions as provided by the Cherenkov detectors, with 25 timing kernels ranging from 180 to 263 ps. Initial reconstruction results with multiple double-Gaussian mixture TOF kernels showed promising characteristics, as they performed *on par* with a 213-ps typical single Gaussian model in terms of contrast recovery but with better BV, due to the higher BGO sensitivity. In addition, we showed that BGO–Cherenkov detectors could provide improved CNR, due to their higher sensitivity.

## Figures and Tables

**TABLE I:** Timing Categories of the Gaussian Mixture Models Used at the BGO-mCh Model. The Values at the First Line Was Used at the BGO-Ch Model.

Kernel	FWHM_*Fast*_ (ps)	FWHM_*Slow*_ (ps)	Abundance Ratio
1	179.8	660.3	0.594
2	193.3	749.9	0.547
3	208.7	784.5	0.527
4	212.8	758.0	0.521
5	224.3	829.5	0.521
6	201.8	772.0	0.575
7	198.2	765.2	0.491
8	214.4	790.4	0.472
9	210.5	776.7	0.446
10	211.4	769.4	0.414
11	209.1	816.8	0.537
12	217.0	817.6	0.486
13	213.8	781.1	0.427
14	233.9	822.5	0.437
15	211.8	791.4	0.385
16	211.8	791.4	0.501
17	229.7	830.1	0.478
18	227.8	822.0	0.421
19	239.5	824.5	0.421
20	257.2	876.1	0.446
21	220.5	817.9	0.501
22	236.6	836.0	0.455
23	240.0	861.9	0.427
24	242.0	879.5	0.419
25	263.8	897.3	0.419
